# Randomized trial to assess the efficacy and safety of xyloglucan for the treatment of acute gastroenteritis in children

**DOI:** 10.1002/fsn3.3688

**Published:** 2023-09-25

**Authors:** Maria Jose Perez‐Garcia, Ana Royuela, Francisco‐Javier Rodriguez‐Contreras, Maria Angeles PandoBravo, Cristina Chiatti, Carmen Ramos, Mario Arana‐Zumaquero, Maria Isabel Gonzalez‐Marcos, Juncal Diaz, Maria Cristina Fresno‐Calle, Ruth García‐Bartolomé, Susana Viver, Serena Villaverde‐Gonzalez, Maria Luz Cilleruelo‐Pascual, Carolina Gutierrez‐Junquera, Alejandro Rasines‐Rodriguez, Alba Manso‐Pérez, Enriqueta Román‐Riechmann

**Affiliations:** ^1^ Department of Pediatrics Hospital Universitario Puerta de Hierro‐Majadahonda Madrid Spain; ^2^ Biostatistics Unit, Hospital Universitario Puerta de Hierro Majadahonda, IDIPHISA CIBERESP Madrid Spain; ^3^ Primary Care Health Center Galapagar Spain; ^4^ Primary Care Health Center Villanueva de la Cañada Spain; ^5^ Primary Care Health Center Brunete Spain; ^6^ Primary Care Health Center Majadahonda Spain; ^7^ Pediatric Gastroenterology Unit, Department of Pediatrics Hospital Universitario Puerta de Hierro‐Majadahonda Madrid Spain

**Keywords:** child, diarrhea, gastroenteritis, randomized controlled trial, xyloglucan

## Abstract

Acute gastroenteritis is one of the most common diseases in children and an important cause of morbidity and mortality worldwide. No specific treatment is available; therefore, management is exclusively symptomatic. Xyloglucan has been approved in Europe as a class IIa medical device for restoration of the physiological functions of the intestinal wall. Our objective was to assess efficacy and safety of xyloglucan for the treatment of acute gastroenteritis in children. We performed a triple‐blind, randomized placebo‐controlled clinical trial in four primary care centers and one continued care hospital center. The study population comprised children with acute gastroenteritis aged >3 months and <5 years. Our primary endpoint was time (in hours) of resolution of diarrhea, defined as the time to resolution of stool consistency (Bristol Stool Form Scale ≤5 or Amsterdam Stool Form Scale B or C) or time until deposition frequency resumes to normality, whichever occurred first. We also recorded intravenous rehydration, hospitalization, stools per day, Vesikari scale, vomiting, relapse, weight loss, drugs prescribed, and adverse events. Eighty children were included in the intention‐to‐treat population (43 xyloglucan and 37 placebo) and 74 (93%) in the per‐protocol population. Time to resolution of diarrhea was similar in both groups with (median, 95% CI) 24, 17–24 h in the xyloglucan group versus 24, 19–24 h in the placebo group, *p* = .680. Significant differences were observed for patients with moderate‐to‐severe diarrhea (Vesikari scale ≥9): xyloglucan group (20 [15–24] h) versus placebo group (85 [51–120] h) (*p* = .04). No other significant differences were found. Xyloglucan can be considered safe and other studies should be performed to confirm the usefulness in patients with moderate‐to‐severe diarrhea.

## Graphical abstract

Xyloglucan is effective and safe in moderate‐to‐severe diarrhea in children 3 months to 5 years.

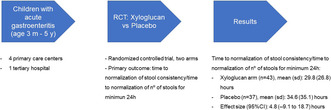

## 1 INTRODUCTION

### 1.1 Background

Acute gastroenteritis is an inflammation of the intestinal mucosa that manifests clinically as diarrhea and vomiting and is generally associated with an intestinal infectious disease. It is one of the most common diseases in children and an important cause of morbidity and mortality worldwide. Major loss of liquids can lead to dehydration, acidosis, and fluid–electrolyte abnormalities. Infants are more vulnerable to gastrointestinal infection and its consequences, namely, dehydration and malnutrition (Guarino et al., 2014, 2020).

### 1.2 Rationale and knowledge gap

Treatment is exclusively symptomatic and aims to correct or prevent dehydration and establish appropriate feeding for nutritional recovery (Guarino et al., 2018). The moderate clinical benefit of some probiotics (*Lactobacillus GG*, *Saccharomyces boulardii*, and *Lactobacillus reuteri*), namely, reduced duration, has been discussed (Szajewska et al., 2020). Drugs are not usually recommended. Antidiarrheal drugs that inhibit motility and modify secretion and adsorbent substances are not indicated in children because their efficacy has not been demonstrated and/or they induce severe adverse effects.

Currently considered agents include racecadotril (specific inhibitor of enkephalinase) (Eberlin et al., 2018; Pienar et al., 2020) smectite, aluminum silicate, and hydrated natural magnesium with absorptive action on toxins, bacteria, and rotavirus. These may be considered for treatment of acute gastroenteritis in children, since they have been shown to reduce total duration of diarrhea, especially in cases associated with rotavirus infection (Florez et al., 2018). The antiemetic ondansetron can also improve the efficacy of oral rehydration in patients with acute gastroenteritis who experience vomiting (Tomasik et al., 2016).

Newly developed mucoprotectors, such as gelatin tannate and xyloglucan (Bueno et al., 2014), can reproduce a muco‐adhesive film or protective sheet in the intestine. Mucus is the first barrier that protects the gastrointestinal tract against microorganisms and antigens, and bacterial invasion is related to the opening of narrow junctions. A recent randomized controlled trial (Kołodziej et al., 2018) did not report differences with respect to the efficacy of gelatin tannate as treatment in children aged under 5 years.

Xyloglucan has been approved in Europe as a class IIa medical device for restoration of the physiological functions of the intestinal wall in the form of capsules for adults and sachets for children. Two randomized open‐label studies in adults (Gnessi et al., 2015) and children (Condratovici et al., 2016) have demonstrated the safety profile of xyloglucan in acute diarrhea. The results of recent placebo‐controlled randomized study with xyloglucan combined with gelose (agar‐agar) in children with acute gastroenteritis showed the combination to be beneficial by reducing the duration of diarrhea (Santos et al., 2021).

### 1.3 Objective

We designed a placebo‐controlled randomized controlled trial (NCT 03357237) to evaluate the efficacy of xyloglucan with gelatin in the treatment of acute gastroenteritis in children.

## 2 METHODS

We performed a triple‐blind randomized parallel phase IV clinical trial in which the random allocation sequence was generated externally using Epidat 4.2 (Consellería de Sanidade, Xunta de Galicia, Spain). This study was registered in ClinicalTrials.gov with the NCT03357237 identifier. Randomization was by 10‐element blocks, to ensure an equal number of subjects in each group after completion of a block. Allocation concealment was achieved by sequentially numbered, opaque, sealed envelopes containing the letter A or B. The person who introduced the sequence into the envelopes was blinded to the treatments represented by A or B. Only the pharmacology laboratory knew the assignation codes, which they opened once the statistical analysis had finished.

### 2.1 Sample size

We hypothesized a superiority trial with 1 day as the superiority limit, starting from 5 days as the average necessary to resolve diarrhea in the placebo group versus 3 days in the xyloglucan group, with a standard deviation for both groups of 2 days. We assumed a power of 80%, significance level of 5%, and proportion of experimental units in the placebo group to the total of 50%. Taking into account an expected 15% dropouts, it would be necessary to recruit 60 patients in the placebo group and 60 patients in the xyloglucan group (a total of 120 patients). Ene3.0 software was used to estimate the sample size.

### 2.2 Setting

Recruitment took place in four primary care centers and one hospital continuing care center.

### 2.3 Inclusion criteria

The study population comprised children with acute gastroenteritis. The inclusion criteria were as follows: clinical acute gastroenteritis defined as a change in stool consistency to loose or liquid according to Bristol Stool Form Scale (6 or 7) or Amsterdam Stool Form Scale (infants) (A) and/or an increase in the frequency of stools (greater or equal to 3/24 h) lasting for no longer than 72 h; and age >3 months and 5 years. Written informed consent was obtained from the parents/guardians before study procedures.

### 2.4 Exclusion criteria

The exclusion criteria were based on those of a study on gelatin tannate with a similar design (Michałek et al., 2016), as follows: treatment with antibiotics, xyloglucan, gelatin tannate, racecadotril, smectite, probiotics, or zinc (including oral rehydration solution containing zinc and/or probiotics) during the previous week; exclusive breastfeeding; chronic gastrointestinal disease (celiac disease, cystic fibrosis, inflammatory bowel disease, food allergy); immunodeficiency; malnutrition (weight/height/length lower than P3 according to WHO standards); severe dehydration; impossibility of follow‐up; known hypersensitivity to gelatin or xyloglucan; absence of informed consent; parents/guardians who do not understand the instructions and requirements of the study; abnormalities in clinical analyses or other medical, social, or psychosocial factors that, in the opinion of the investigator, could have a negative effect.

### 2.5 Patient and public involvement

Participants were enrolled by pediatricians from the emergency department and primary care after verification of the inclusion and exclusion criteria. Once the informed consent had been signed, pediatricians assigned participants to the intervention by opening the envelope.

Patients were not involved in the design of this study; however, children's parents were involved in the conduction of the study, as they were provided with the phone number of their corresponding pediatrician to ask any question regarding the study.

### 2.6 Intervention

Participants and professionals who assigned participants to interventions were blinded to the treatment. The composition of the placebo was identical to that of the product, with maltodextrin instead of xyloglucan and gelatin. The flavor—vanilla‐strawberry—was the same for both (Laboratorios Ferrer, Barcelona, Spain [both products]). The statistician who analyzed the data was also blinded. Allocation sequence was concealed with opaque envelopes and was only known when the participant accepted to participate in the study. Patients were assigned to one of two groups: the intervention group, which received oral rehydration solution (ORS) and xyloglucan with gelatin, and the control group, which received ORS and placebo.

Xyloglucan was administered at an age‐dependent dose (100 mg/8 h for age <3 years and 200 mg/8 h for age 3 and 4 years); both study products were taken three times per day for 5 days. The medication was started as soon as the diagnosis was made, it was confirmed that the patient met inclusion criteria, the parents were informed and the informed consent was signed.

Treatment was standard in acute gastroenteritis in both groups with respect to clinical evaluation, complementary examinations, rehydration, and feeding (ESPGHAN 2014 recommendations) (Guarino et al., 2018). At inclusion, parents/guardians received a diary to record symptoms, as well as stool number and consistency (Bristol or Amsterdam Stool Form Scales, also provided to the parents). Adverse events during the intervention and follow‐up were also recorded. A stool sample was taken for the microbiology work‐up (rotavirus and adenovirus antigens and standard stool culture).

Children were followed up by telephone on days 2, 5, and 14 after the initial visit and in person on day 7. Parents/guardians recorded administration of the product and the number of sachets left unused at the end of the study. Administration of <75% of the dose was considered nonadherence.

### 2.7 Outcomes

#### 2.7.1 Primary outcome

Time (in hours) of resolution of diarrhea, defined as the time to resolution of stool consistency (achieving a Bristol Stool Form Scale ≤5, or an Amsterdam Stool Form Scale equals to B or C) or time until deposition frequency resumes to normality, compared with the period before the onset of diarrhea for 24 h, whichever occurred first.

#### 2.7.2 Secondary outcomes

Need for intravenous rehydration, need for hospitalization, mean liquid stools per day, severity of diarrhea (Vesikari scale) (Schnadower et al., 2013), vomiting, weight gain, recurrence of diarrhea (48 h after the intervention), use of concomitant medication, and adverse effects.

The study protocol was approved by the corresponding Hospital Ethics Committee.

### 2.8 Statistical analysis

Analyses were performed on an intention‐to‐treat (ITT) basis and were repeated for the per‐protocol (PP) population. The ITT population was defined as that receiving at least one dose.

The descriptive analysis was performed using absolute and relative frequencies for categorical variables and mean and standard deviation (SD) or median and interquartile range (IQR) for numerical data according to the distribution of data across the treatment arms.

The primary outcome was tested using the Mann–Whitney test, since time variables are rarely normally distributed. We also performed a linear regression analysis to investigate effect size. If any baseline variable was not equally distributed between the two arms of treatment, we adjusted for these variables using a multivariable linear regression. We performed a subgroup analysis in patients with severe diarrhea (Vesikari scale ≥9) for the primary outcome. Secondary outcomes were compared using the chi‐square test, *t*‐test, or Mann–Whitney test, as applicable.

A *p*‐value < .05 was considered statistically significant. Stata 15 was used for the statistical analyses (StataCorp. 2017; Stata Statistical Software: Release 15; StataCorp LLC).

The safety analysis was performed by collecting all adverse events observed during the 10–14 days after initiation of treatment. The need for complementary testing was investigated using the clinical history and physical examination. Further information can be found in the Supplementary Statistical Analysis Plan (SAP) file.

Adverse events observed during the 10‐ to 14‐day period were recorded in the case report folder by the parents/guardians and by the investigators at each of the scheduled visits. The progress of adverse events reported at previous visits was also recorded. Safety and adverse effects were analyzed using contingency tables and compared using the chi‐square or Fisher's exact test.

## 3 RESULTS

**FIGURE 1:**
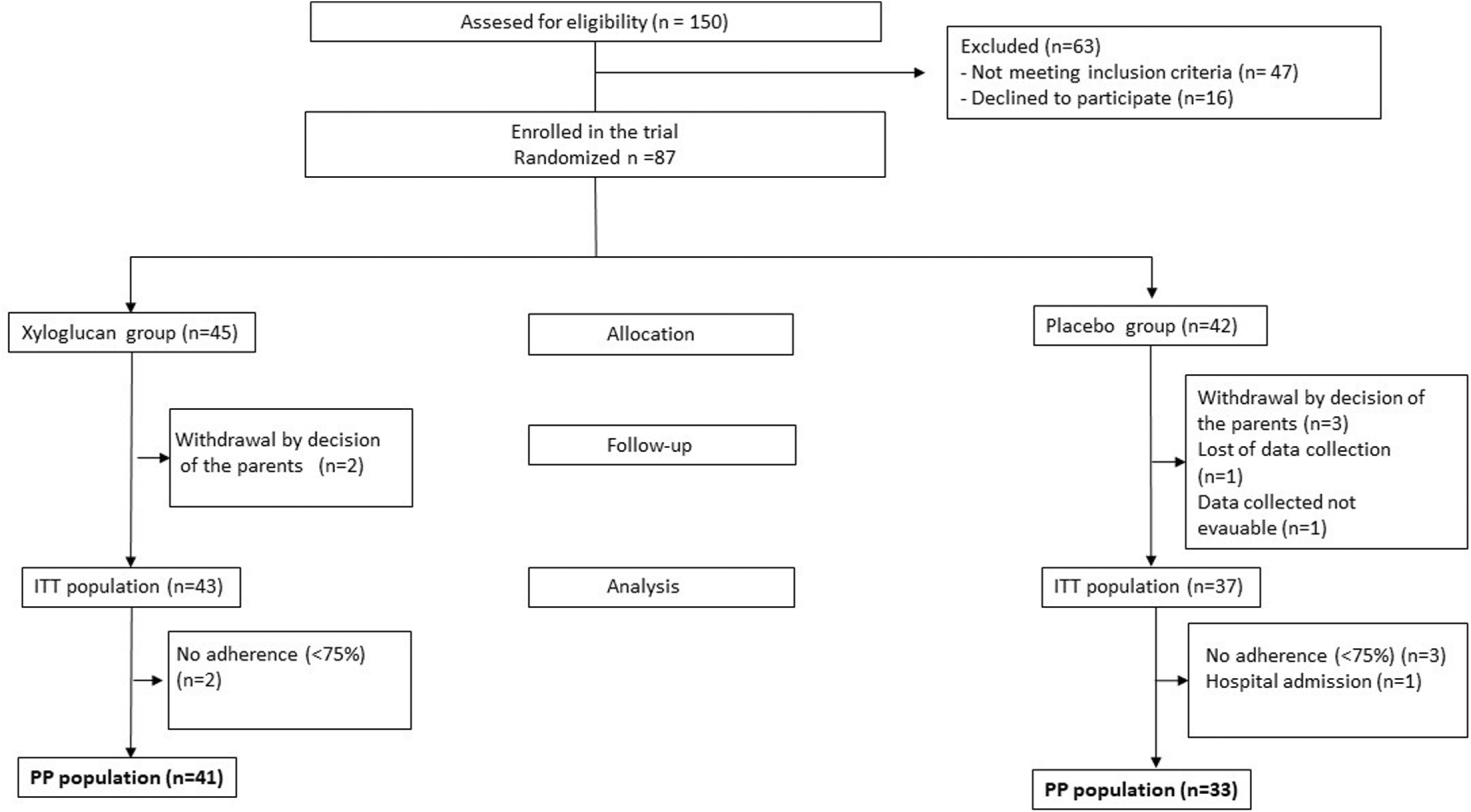
Study flowchart. ITT, Intention to treat; PP, Per protocol.

Overall, 150 children with acute gastroenteritis who presented for treatment between March 2018 and February 2019 were assessed for eligibility; 87 were enrolled in the study and randomly assigned to one of the two study groups: 45 to the xyloglucan group and 42 to the placebo group. Thirteen children were lost to follow‐up (Figure 1). This work has followed the CONSORT statement (Appendix S1).

Eighty children were included in the ITT population and analyzed (43 for xyloglucan and 37 for placebo) and 74 (93%) in the PP population, in which the intervention was completed (Figure 1).

Baseline demographic and clinical characteristics (ITT population) are shown in Table 1. Both groups were comparable in these characteristics at study entry.

**TABLE 1:** Baseline demographic and clinical characteristics (ITT population).

Characteristics	Xyloglucan group (*n* = 43)	Placebo group (*n* = 37)
Age, months, mean (SD)	20.1 (13.3)	24.4 (15.2)
Age, months, median (IQR)	14 (10–29)	21 (13–34)
Sex, male/female, *n*	24/19	14/23
Dehydration level before enrolment, *n* (%)		
No dehydration	25 (58)	20 (56)
Mild	18 (42)	16 (44)
Moderate	0 (0)	0 (0)
Severe	0 (0)	0 (0)
Vesikari scale, median (IQR)	6 (5–8)	6 (5–8)
Fever (≥38°C), *n* (%)	18 (42)	13 (35)
Vomiting, *n* (%)	20 (48)	20 (54)
Abdominal pain, *n* (%)	28 (65)	21 (57)
Blood in stool, *n* (%)	0 (0)	3 (8)
Etiology of acute gastroenteritis		
Rotavirus, *n* (%)	6 (15.4)	1 (3.5)
Adenovirus, *n* (%)	0 (0.0)	1 (3.5)
Campylobacter, *n* (%)	2 (5.1)	3 (10.3)
Unknown etiology, *n* (%)	35 (79.5)	33 (82.7)
Current breastfeeding, *n* (%)	9 (21)	10 (27)
Nursery school, *n* (%)	28 (65.1)	32 (86.5)

### 3.1 Primary and secondary outcomes

Time to resolution of diarrhea after randomization was similar in both groups, with a median of 24 (p25–p75: 14–47) h in the xyloglucan group and 24 (14–48) h in the placebo group. No significant differences were recorded between the groups (*p* = .683, Mann–Whitney).

Diarrhea took on average 3.5 h more to normalize in the placebo arm than those receiving the active treatment (95% CI, −11.1 to 18.1; *p* = .634 [linear regression value adjusted for vomiting, age in months, fever, and nursery school]) (Table 2).

**TABLE 2:** Primary outcome.

Primary outcome analysis: hours to normal stool consistency or number for a minimum of 24 h, whichever occurs first (*n* = 80)				
	Mean (SD)	Median (IQR)	Univariable regression (95% CI) (*p*‐value = .493)	Adjusted regression^a^ (95% CI) (*p*‐value = .634)
Xyloglucan	29.8 (26.8)	24 (14–47)	4.8 (−9.1 to 18.7)	3.5 (−11.1 to 18.1)
Placebo	34.6 (35.1)	24 (14–48)		

Subgroup analysis for participants with moderate‐to‐severe diarrhea (Vesikari scale ≥9) for the primary outcome (*n* = 15)				
	Mean (SD)	Median (IQR)	Univariable regression (95% CI) (*p*‐value = .010)	Adjusted regression^a^ (95% CI) (*p*‐value = .017)
Xyloglucan (*n* = 10)	24.0 (21.1)	20 (15–24)	62.8 (17.7–107.9)	65.6 (14.7–116.4)
Placebo (*n* = 5)	86.8 (61.0)	85 (51–120)		

^a^ Adjusted for vomiting, age (months), fever, and nursery school.

In a not‐planned sensitivity analysis, significant differences were observed when analyzing patients with moderate‐to‐severe diarrhea (baseline Vesikari scale ≥9, 10 patients in the xyloglucan group vs. 5 in the placebo group), with 65.6 h more on average to normalize diarrhea in patients assigned to the placebo arm (95% CI, 14.7–116.4; *p* = .017). The median time to resolution was 20 (15–24) h in the xyloglucan group and 85 (51–120) h in the placebo (*p* = .049, Mann–Whitney) (Table 2). No other significant differences were found.

The number of watery stools per day was similar in both groups throughout the study period (Table 3).

**TABLE 3:** Secondary outcomes.

Outcomes	Xyloglucan group (*n* = 43)	Placebo group (*n* = 37)	*p*‐value
Need for intravenous rehydration, *n* (%)	0 (0.0)	2 (5.4)	.123
Number of watery stools per day^a^ (median [IQR])			
Day 1	3 (2–4)	2 (1–4)	.261
Day 2	2 (1–3)	2 (1–3)	.455
Day 3	2 (1–2)	2 (1–3)	.822
Day 4	2 (1–2)	2 (1–3)	.200
Day 5	1 (1–2)	2 (1–3)	.302
Vomiting, *n* (%)	16 (37.2)	13 (35.1)	.847
Weight gain, kg (median [IQR])	0.1 (−0.2 to 0.3)	0.0 (−0.1 to 0.2)	.267
Recurrence of diarrhea (frequency) (48 h after intervention), *n* (%)	16 (38.1)	12 (36.4)	.878
Recurrence of diarrhea (consistency) (48 h after intervention), *n* (%)	12 (28.6)	11 (31.4)	.785
Severity of diarrhea according to Vesikari Scale (maximum reached) (median [IQR])	7 (5–9)	8 (6–9)	.439
Need for hospitalization in outpatients, *n* (%)	0 (0)	1 (2.7)	.278
Adverse events, *n* (%)	1 (2.3)	1 (2.7)	1.000

^a^ According to the Bristol Stool Form (BSF) Scale or Amsterdam Stool Form (ASF) Scale (on BSF scale, numbers 2, 3, 4, and 5; on ASF scale, letters B or C).

The frequency of vomiting was similar in both groups (16 patients in the xyloglucan group vs. 13 in the placebo group). The same is true of weight loss, need for intravenous rehydration, and recurrence of diarrhea 48 h after the intervention, both for consistency and increased frequency of stools, although no significant differences were found in either group (Table 3). The median Vesikari scale score was similar among the groups (seven in the xyloglucan group and eight in the placebo group) (Table 3).

Adverse effects were similar in both groups. One patient vomited after taking the product in the xyloglucan group (30% of doses), and one patient experienced rash, probably in association with the viral infection causing gastroenteritis (sudden‐onset rash). One case of vomiting associated with the product was recorded in the placebo group (the patient did not finish the dose).

Six of the 80 patients did not complete treatment, since they received <75% of the recommended doses. Therefore, the PP population comprised 74 participants. The results were similar for this population (data not shown). In the case of the patients who did not complete treatment, the reason for rejection was taste.

## 4 DISCUSSION

### 4.1 Key findings

We found no differences between the arms for the main outcome, which was time to resolution of diarrhea. However, in the subgroup of patients with more severe diarrhea (Vesikari scale ≥9), the results might reveal the benefit of xyloglucan, with significant shortening of the duration of diarrhea.

### 4.2 Strengths and limitations

This study is designed as a triple‐blinded, randomized, placebo‐controlled trial, corresponding to the highest level in the evidence pyramid. The study answers a relevant clinical question about the effectiveness and safety of xyloglucan. Completeness of follow‐up was available in 85% of the participants. One of the main limitations was that the designed sample size was not reached, and it implied a lack of statistical power. We found several difficulties during the recruitment phase because, on the one hand, the attended population showed a very high level of health education, and they only visit their doctor in the more extreme cases (80% of our patients had mild diarrhea). Most of the cases presented at the ED with more than 72‐h diarrhea evolution, so therefore, several patients were discarded because not accomplishment of inclusion criteria. Likewise, many of the patients had already started home treatment with probiotics, which also excluded them.

### 4.3 Comparison with similar researches

While pediatric gastroenteritis is currently a self‐limiting condition in our setting, it is still considered important to obtain a safe and effective drug that affects the natural history and shortens the process.

Our results are not comparable to those reported for adults. While we found the product to be efficacious in moderate‐to‐severe diarrhea, no improvement was observed in the population as a whole, mostly mild cases. A multicenter randomized open‐label efficacy and safety study compared xyloglucan/gelatin with *Saccharomyces boulardii* and smectite (Gnessi et al., 2015) in 150 adults and found more rapid onset in terms of reduced number of stools (Bristol 6–7) at 6 h of treatment in the group treated with xyloglucan (*p* = .031) and greater efficiency, seen as a reduction in the percentage of patients with nausea and abdominal pain. We found no significant differences in the reduction for other accompanying digestive symptoms. The mild nature of the symptoms in most cases of gastroenteritis in our study could explain the absence of such beneficial results.

A multicenter open‐label randomized trial comparing the efficacy and safety profile of xyloglucan and ORS with that of ORS alone (Condratovici et al., 2016) included 36 patients with acute gastroenteritis aged 3 months to 12 years who were randomly assigned to xyloglucan + ORS or ORS alone for 5 days. Symptoms and adverse events were evaluated at 2 and 5 days. Patients treated with xyloglucan and ORS progressed better, with a significant decrease in the number of stools 6 h after starting treatment (0.11 vs. 0.44) and persistence of diarrheic stools (Bristol 6–7) on the 3rd day of treatment in 44% of the ORS group and 11% of the xyloglucan + ORS group. The patients in this study were significantly older than in ours (range, 3 months–12 years, mean 4.3 years). Similarly, only the Bristol scale was used. On comparing the results, we believe that inappropriate interpretation of the graphic section of the Amsterdam scale in patients who used a diaper could have negatively affected the findings, given that in our study, the median age was 14 and 21 months, respectively, in the xyloglucan and placebo groups.

The results of a recent double‐blind, placebo‐controlled randomized clinical trial in children aged 3 months to 14 years with acute gastroenteritis treated with xyloglucan and gelose (Santos et al., 2021) showed a reduction in total diarrheic stool (Bristol 6–7) in the xyloglucan and gelose group. The difference between this group and the placebo group was statistically significant, and rapid onset of action was observed 6 h after initiation of treatment. These results are not comparable with those of our study owing to differences in the populations included, with a median age of 5.2 years and poor representation of nursing infants, the population that is most vulnerable to the complications of gastroenteritis. Similarly, measurement of the outcomes was not comparable (reduction in total number of stools per group).

With respect to studies published with other antidiarrheal drugs approved for children, a 2015 meta‐analysis of the efficacy and safety of smectite for treatment of gastroenteritis in children concluded that the drug was useful in that it shortened the duration of diarrhea. However, the GRADE evidence generated was of low quality, since most of the trials were open‐label, with the result that there was a high possibility of publication bias. This meta‐analysis included a total of 13 randomized clinical trials with 2164 patients aged 1–60 months (Das et al., 2015).

The use of racecadotril in children was examined in a meta‐analysis, where after evaluation of efficacy compared with placebo and other antidiarrheal agents, the authors concluded that racecadotril is more efficacious than other treatments, except for loperamide, and has a tolerability similar to that of placebo and better than that of loperamide. These findings support the use of racecadotril in the treatment of acute diarrhea in children (Eberlin et al., 2018).

A recent study with another antidiarrheal product (gelatin tannate) that was similar in design to ours (sample size) (Kołodziej et al., 2018) included progress of gastroenteritis of more than 5 days. The authors did not find gelatin tannate to be efficacious, although they did suggest that early administration of the product at onset of symptoms could increase efficacy. In our study, one of the inclusion criteria was that the presentation of diarrhea was acute and duration short (<72 h) in order to avoid normal early natural resolution in mild cases. No significant differences were found, despite the early administration of xyloglucan in the case group (mostly mild). Of note, in those cases of moderate‐to‐severe diarrhea (20% of participants), early administration showed xyloglucan to be efficacious.

## 5 CONCLUSION

The results of this triple‐blind, placebo‐controlled trial confirm that xyloglucan administered during the first 72 h of onset of acute gastroenteritis in patients aged 3 months to 5 years is a safe antidiarrheal agent. More prospective studies must be performed to confirm the effectiveness in the more severe cases.

## AUTHOR CONTRIBUTIONS

**Ana Royuela:** Data curation (lead); formal analysis (lead); methodology (lead); software (lead); supervision (equal); validation (equal); visualization (equal); writing – original draft (equal); writing – review and editing (equal). **Maria Jose Perez‐Garcia:** Conceptualization (equal); data curation (equal); methodology (equal); project administration (equal); resources (equal); validation (equal); visualization (equal); writing – original draft (equal). **Francisco‐Javier Rodriguez‐Contreras:** Data curation (equal); investigation (equal); resources (equal); supervision (equal); writing – review and editing (equal). **Maria Angeles PandoBravo:** Data curation (equal); investigation (equal); project administration (equal); resources (equal); supervision (equal); writing – review and editing (equal). **Cristina Chiatti:** Data curation (equal); investigation (equal); project administration (equal); resources (equal); supervision (equal); writing – review and editing (equal). **Carmen Ramos:** Data curation (equal); investigation (equal); project administration (equal); resources (equal); supervision (equal); writing – review and editing (equal). **Mario Arana‐Zumaquero:** Data curation (equal); investigation (equal); project administration (equal); resources (equal); supervision (equal); writing – review and editing (equal). **Maria Isabel Gonzalez‐Marcos:** Data curation (equal); investigation (equal); project administration (equal); resources (equal); supervision (equal); writing – review and editing (equal). **Juncal Diaz:** Data curation (equal); investigation (equal); project administration (equal); resources (equal); supervision (equal); writing – review and editing (equal). **Maria Cristina Fresno‐Calle:** Data curation (equal); investigation (equal); project administration (equal); resources (equal); supervision (equal); writing – review and editing (equal). **Ruth García‐Bartolomé:** Data curation (equal); investigation (equal); project administration (equal); resources (equal); supervision (equal); writing – review and editing (equal). **Susana Viver:** Data curation (equal); investigation (equal); project administration (equal); resources (equal); supervision (equal); writing – review and editing (equal). **Serena Villaverde‐Gonzalez:** Data curation (equal); investigation (equal); project administration (equal); resources (equal); supervision (equal); writing – review and editing (equal). **Maria Luz Cilleruelo‐Pascual:** Data curation (equal); investigation (equal); resources (equal); visualization (equal); writing – review and editing (equal). **Carolina Gutierrez‐Junquera:** Data curation (equal); investigation (equal); project administration (equal); resources (equal); writing – review and editing (equal). **Alejandro Rasines‐Rodriguez:** Data curation (equal); investigation (equal); resources (equal); writing – review and editing (equal). **Alba Manso‐Pérez:** Data curation (equal); investigation (equal); resources (equal); visualization (equal); writing – review and editing (equal). **Enriqueta Román‐Riechmann:** Conceptualization (equal); funding acquisition (equal); project administration (equal); supervision (equal); validation (equal); writing – original draft (equal); writing – review and editing (equal).

## FUNDING INFORMATION

This work was supported by Laboratorios Ferrer, Barcelona, Spain.

## CONFLICT OF INTEREST STATEMENT

All authors have completed the ICMJE uniform disclosure form. The authors have no conflicts of interest to declare. The authors have completed the CONSORT reporting checklist.

## ETHICS STATEMENT

The authors are accountable for all aspects of the work in ensuring that questions related to the accuracy or integrity of any part of the work are appropriately investigated and resolved. The trial was conducted in accordance with the Declaration of Helsinki (as revised in 2013). The study was approved by the Ethics Committee of Hospital Universitario Puerta de Hierro (CPMP/ICH/135/95) and informed consent was taken from all individual participants.

## Supporting information


Appendix S1

## Data Availability

Data are available upon reasonable request.
